# Semantic Feature Extraction Using SBERT for Dementia Detection

**DOI:** 10.3390/brainsci12020270

**Published:** 2022-02-15

**Authors:** Yamanki Santander-Cruz, Sebastián Salazar-Colores, Wilfrido Jacobo Paredes-García, Humberto Guendulain-Arenas, Saúl Tovar-Arriaga

**Affiliations:** 1 Facultad de Ingeniería, Universidad Autónoma de Querétaro, Queretaro C.P. 76010, Mexico; syamanki@gmail.com (Y.S.-C.); wilfrido.paredes@uaq.mx (W.J.P.-G.); 2 Centro de Investigaciones en Óptica, Leon C.P. 37150, Mexico; sebastian.salazar@cio.mx; 3 Departamento de Geriatría, Instituto Mexicano del Seguro Social, San Juan del Rio C.P. 76800, Mexico; drguendulaingeriatria@gmail.com

**Keywords:** dementia, SBERT, semantic analysis, syntax analysis, NLP feature extraction

## Abstract

Dementia is a neurodegenerative disease that leads to the development of cognitive deficits, such as aphasia, apraxia, and agnosia. It is currently considered one of the most significant major medical problems worldwide, primarily affecting the elderly. This condition gradually impairs the patient’s cognition, eventually leading to the inability to perform everyday tasks without assistance. Since dementia is an incurable disease, early detection plays an important role in delaying its progression. Because of this, tools and methods have been developed to help accurately diagnose patients in their early stages. State-of-the-art methods have shown that the use of syntactic-type linguistic features provides a sensitive and noninvasive tool for detecting dementia in its early stages. However, these methods lack relevant semantic information. In this work, we propose a novel methodology, based on the semantic features approach, by using sentence embeddings computed by Siamese BERT networks (SBERT), along with support vector machine (SVM), K-nearest neighbors (KNN), random forest, and an artificial neural network (ANN) as classifiers. Our methodology extracted 17 features that provide demographic, lexical, syntactic, and semantic information from 550 oral production samples of elderly controls and people with Alzheimer’s disease, provided by the DementiaBank Pitt Corpus database. To quantify the relevance of the extracted features for the dementia classification task, we calculated the mutual information score, which demonstrates a dependence between our features and the MMSE score. The experimental classification performance metrics, such as the accuracy, precision, recall, and F1 score (77, 80, 80, and 80%, respectively), validate that our methodology performs better than syntax-based methods and the BERT approach when only the linguistic features are used.

## 1. Introduction

Dementia, defined in the Diagnostic and Statistical Manual of Mental Disorders (DSM 5) [1] as a major neurocognitive disorder, is a condition with etiological subtypes in which different levels of dysfunction are recognized. It affects older adults, showing its first signs at the age of 60 years old. Alzheimer’s disease (AD), one of the most prevalent subtypes, registers 50 million diagnosed people, a number that is expected to double by 2050, according to statistics reported by the Alzheimer’s Association [2]. The characteristic symptoms of AD are linguistic deficits in oral and written expression, difficulty in motor planning, and impaired sensory and spatial perception [1]. This is reflected in the aspects of the intellectual functions and processes, such as attention, memory, cognition, decision making, planning, reasoning, judgment, perceptual comprehension, language, and the visuospatial function [3]. The standard diagnostic procedure corroborates deficits in two or more cognitive areas with neuropsychological tests and physical and neurological examinations. The application of cognitive assessment follows them. The Montreal Cognitive Assessment (MOCA) and the Mini-Mental State Examination (MMSE) are the most common cognitive assessment methods. In addition, medical tests are performed to rule out other causes of dementia-like symptoms [4]. The diagnosis of dementia may be delayed because of three reasons: first, the need for a third person to identify the signs: second, the similarity of the signs to other disorders, such as major depression and other types of neurological diseases; and third, the social stigma that detracts from the treatment of diseases related to mental conditions that require specialized medical care [5]. Furthermore, not all people have access to mental health services because of socioeconomic situations. This situation arises mainly in developing regions, such as Latin America, where the prevalence of dementia is estimated at 11% in the adult population [6].

Dementia is an incurable condition, and the treatment focuses on delaying deterioration. Early detection is the most effective measure to halt the progression of cognitive impairment [7]. It is essential to have clinical instruments in order to obtain the most information concerning diagnostic screening. The early cognitive markers of dementia focus on episodic memory and orientation [8]; however, recent research finds that semantic memory and language skills can be sensitive tools for early diagnosis [9].

The global coherence of the spontaneous speech of AD patients shows semantic comprehension and memory loss alterations [4]. Semantic comprehension alterations include errors when naming objects, or actions resulting in the incorrect categorical naming of entities, and a failure to organize semantic knowledge structures, or a disconnection between the hierarchical organization of knowledge and verbal production [10]. Memory loss alterations are reflected in the restricted vocabulary of AD patients and their difficulties in finding appropriate words for sentences [11]. Neuropsychological evaluations are performed to detect such alterations and deficits. One of the most commonly used neuropsychological evaluations is the cookie thief test [12], which describes a picture that provides valuable information about the oral discourses of patients. The cookie thief test enables linguistic analyses from various perspectives, where grammar, linguistic expression, the quality of the conveyed information, the topic mastery, and the narrative organization can be explored and identified as areas of impairment in the preclinical stages of AD.

Natural language processing (NLP) methods have performed well in feature extraction and pattern search tasks in the linguistic context for tasks related to the problem addressed in this paper, such as those described in [13,14,15]. As previously stated, this paper proposes using NLP to extract linguistic features from a previously preprocessed text from the Pitt Corpus database provided by DementiaBank [16]. Text preprocessing consists of extracting the patient’s speech and converting it into plain text. The incomplete words and grammatical errors are then captured and used as features. In a complementary sense, additional features of different natures are incorporated, such as: the vocabulary length; the lexical diversity; the number of keywords; demographic information, such as the age and years of education; and the features obtained from BERT Siamese networks (SBERT) in the semantic search for main ideas, ground-truth semantic similarities, and main idea counts.

Finally, support vector machine (SVM), K-nearest neighbors (KNN), random forest, and artificial neural networks (ANNs) were incorporated into the methodology in the AD classification stage. To measure the performance of our method, we applied the metrics commonly encountered in the literature, such as the accuracy, precision, recall, and F1 score. Since the dataset’s cardinality is reduced (550) to have the least possible biased view of the performance of our proposed method, the results are presented as the averages of a 10-K-fold cross validation, with k = 10. The obtained results show that the best classification model is based on SVM, with the polynomial kernels reaching 77, 80, 80, and 80% of the accuracy, precision, recall, and F1 score, respectively.

The contents of this paper are presented as follows: Section 2 provides a review of the related work; Section 3 describes the materials and methods on which this work is based, and shows the results obtained; Section 4 discusses and analyzes the performance of the proposed methodology; and finally, Section 5 presents our conclusions.

## 2. Related Work

NLP algorithms based on deep learning present a vast area of opportunity to make forays into the healthcare domain because of their ability to analyze large amounts of multimodal data through computational processing [17,18]. However, these NLP methods require a large amount of data to perform well. Unfortunately, clinical databases in the study of dementia present scarcities and limited access to this type of information. Faced with this drawback, Masrani et al. [19] constructed a corpus of several thousand blog entries, some from people with dementia, and some from control persons. They used this dataset to design an AD classifier on the basis of random forest and KNN, and the neural networks achieved accuracies of 84%. The linguistic features of AD patients have been used for the classification of this disease. It is important to mention that the datasets are formed by the speech data in the works described below. However, the analysis is text-based since, during the evaluation, the oral utterances are recorded and then transcribed under a protocol in which most of the information about the subject’s oral performance, such as pauses and cadence, is maintained. The state-of-the-art methods carried out can be divided into two stages: the first stage requires human intervention to perform the evaluations and preprocess the data; and the second stage consists of automatic methods, with the corpus as input, and the classification labels as output.

Roak et al. [20] have explored pause counting and syntactic complexity analyses extracted from audio transcripts built manually from 74 recordings created during neuropsychological examinations of those who were assigned as healthy, and those diagnosed with mild cognitive impairment, by which they performed a classification model on the basis of a support vector machine (SVM) with a second-order polynomial kernel, obtaining an accuracy rate of 86% from their classification. Other studies propose to strengthen the classification models by adding demographic information [21,22], electronic medical recordings (EMR) [23], and medical measures, such as MMSE scores [24]. Karlekar et al. [25] propose a classification method of three artificial neural models based on convolutional neural networks (CNNs), long short-term memory (LSTM-RNNs), and their combination, in order to distinguish between the language samples of AD and control patients, achieving an 84.9 % accuracy in the Pitt Corpus database, provided by DementiaBank, composed of 243 controls and 307 AD patients. Solis-Rosas et al. [26] performed an in-depth syntactic analysis, including pauses, filler words, formulated words, restarts, repetitions, incomplete utterances, and fuzzy speech. They performed two automatic classification methods, one using a 3-layer ANN, and a second one using an SVM with a polynomial kernel. Their maximum classification rate reached 86.42% accuracy on the Carolina Conversations Collection, which contains 256 samples from the conversations of dementia patients and healthy people [27]. In another study, Eyigoz et al. [28] use linguistic variables with clinical and demographic variables in their prediction models. Extracted from the Framingham heart study database [29], their study achieved 70% accuracy.

In recent years, some research has been performed in which the use of the acoustic features extracted from the neuropsychological test’s audio records, in addition to the linguistic features, has been proposed [30,31,32,33]. These works use the ADReSS Challenge database [34], which was formed to perform two main tasks: the first is an MMSE score regression task that is used to create a model to infer the subject’s MMSE score, on the basis of the speech production during a neuropsychological assessment; and the second is an AD classification task, where the production of a model to predict the label of “AD” or “non-AD” for a speech session is required. The dataset contains samples from 78 non-AD subjects and 78 AD subjects. Mostly, an SVM model, a random forest model, and some neural network architectures were employed to try to solve these tasks, achieving accuracy rates of 77% and 77%, a recall of 76%, and an F1 score of 77%.

Balagopalan et al. [35] explore, for the first time (to the best of the authors’ knowledge), the use of semantic features as the average cosine distance between the utterances and the average cosine distance between the 300-dimensional word2vec utterances and the picture content units, in addition to the acoustic and linguistic features and the text classification by BERT. After the classification stage, they obtained an accuracy of 81%, a precision of 83%, a recall of 79%, and an F1 score of 81%. In the case of the classification by BERT, the metrics achieved were as follows: 83% accuracy; 86% precision; 79% recall; and 83% F1 score.

Through this brief overview of some of the projects conducted on the search for dementia patterns using NLP techniques, we can observe that mainly lexical and syntactic linguistic features were deployed. However, it remains to be explored how semantic elements provide helpful information when performing the automated detection of dementia. In this work, the extraction and study of the semantic features to evaluate the presence of dementia is carried out. Hence, lexical, syntactic, and semantic analyses, based on NLP, were performed.

## 3. Materials and Methods

This section presents our proposed methodology to classify dementia in its early stages. The dataset format, the feature extraction-selection process, and the classification methods are described.

### 3.1. Dataset

This study used data from the Pitt Corpus [16,36], which consists of audio recordings and transcripts, demographic data, and the MMSE test results collected as part of the protocol administered by the Alzheimer’s and Related Dementias Study at the University of Pittsburgh School of Medicine. The participants included 243 healthy controls (HC), 307 people with probable and possible Alzheimer’s disease (AD), and other dementia diagnoses, such as vascular dementia and Parkinson’s, and all of them were native speakers of English. Table 1 summarizes the demographic data matched by the HC and AD groups.

**Figure 1:**
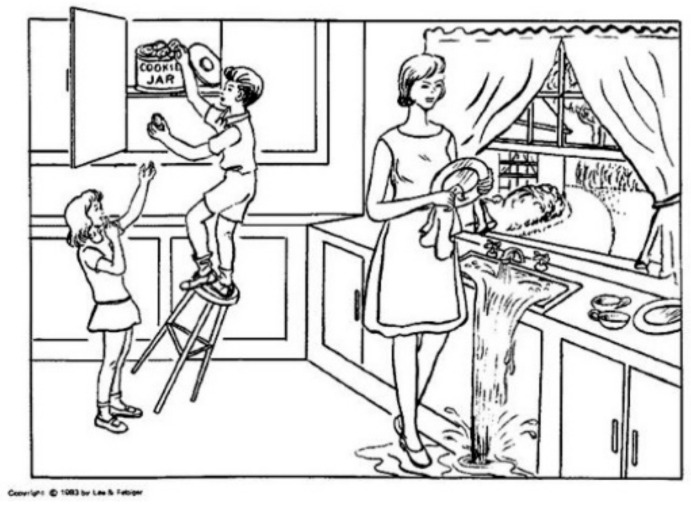
Image used in cookie thief test from the Boston aphasia diagnostic test [11].

The Pitt Corpus contains the manual transcripts from the cookie, fluency, and recall tasks. Each speech sample recording was transcribed manually at the word level, following the TalkBank CHAT (Codes for the Human Analysis of Transcripts) protocol [37]. For this study, we focus on the cookie thief test, since it provides a standardized test that has been used in various studies in the past [10]. The patients were given the cookie thief picture during the evaluation and were asked to describe everything. The picture consists of a familiar domestic scene, as is shown in Figure 1, the description of which requires the use of the basic essential vocabulary learned in childhood and the contrasting of characters and places.

The aim of applying this cookie thief evaluation is to gather information regarding the existing communication and attention deficits in patients. We are interested in highlighting the use of lexical, syntactic, and semantic features as indicative of cognitive impairment.

### 3.2. Lexical Analysis

“Lexical analysis” refers to the validation of words from a linguistic point of view, which is derived from the lexemes of the language in which the text has been written, which, in this case, is English [38]. We calculated four metrics that capture the lexical complexity of a text:

- Word truncation: People with dementia often forget the word and struggle to finish it. Each word is checked against a dictionary to make sure the words are complete. In this way, the number of truncated words is counted and stored;
- Vowel repetition: Bardilalia is a language disorder that results in the slowing of speech production and great difficulty in articulating words. Commonly, multiple blocks appear during the speech (stuttering), as well as certain prolongations or repetitions of the sounds of the words. The search for the vowel addition to the correct spelling of the words was performed by comparing the word extracted from the transcription with respect to its dictionary entry;
- Vocabulary length: The tokenization of the text has been carried out, an operation in which the separation of the words used within the corpus is performed. From the tokenization of words, it is feasible to perform a word bag model in which all the words used throughout the text are identified. After identifying these words, the frequencies with which they appear in the text are counted. It is then possible to measure the vocabulary length (VL);
- Lexical diversity: Since the vocabulary of people with dementia in the middle and late stages tends to be very poor [10], the lexical diversity (LD) is an important metric in this study, as it indicates the amount of vocabulary used during the description;
- It is possible to calculate the lexical diversity from the following formula, where the text length (TL) is divided by the number of vocabulary words used, the vocabulary length (VL):
  LD = TL/VL(1)
  It is important to clarify that the calculations of the spelling and typing errors are not perfect, since some of these errors may not be identified in the dictionary.

### 3.3. Syntactic Analysis

“Syntactic analysis” refers to the use of words within a sentence. It is necessary to have concrete knowledge of the grammatical rules, on the basis of which it is possible to decompose the sentence (tokenize), generating a syntactic tree that allows us to move through its structure.

It is common for patients diagnosed with AD to find it very difficult to maintain a conversation because of their constant difficulties finding the right word, or because of substituting the wrong one and sometimes forgetting that they have already said that word [34].

A pattern that is indicative of dementia is the tendency to shorten and structure grammatically incorrect sentences, and to perform the word repetition mentioned in a short period, since the episodic memory is in the process of deteriorating. A syntactic analysis of the patient’s narrative production is required in order to obtain the grammatical and sentence structural errors.

To do this, we make use of the API Language Tool, which is based on the part-of-speech tagger (POS-tagger), where the parts of speech are assigned to each word (and other tokens), such as noun, verb, and adjective [39]. The grammatical errors made during the description are recollected, as well as the number of repeated words in the same sentence.

### 3.4. Semantic Analysis

There are two approaches to semantics. The first one refers to the meaning that words have by themselves. The second one considers the meanings acquired by their use in a given circumstance. A semantic analysis needs to work on the syntactic structure at the computational level because this establishes a relationship that gives meaning to a sentence through the words that precede or proceed it. Unfortunately, semantic technology processes ignore the influences of the context and the speaker’s intentions; however, it is possible to contextualize the text mathematically.

We have implemented three methods that will help us make sense of the text and evaluate whether the evaluation objective is achieved within the description by highlighting the image features of interest: (1) A keyword search; (2) A similarity calculation to find an approximation to the text’s central ideas; and (3) A similarity calculation concerning the description determined as a ground truth. This was performed in order to have a broad search threshold for the test subjects’ answers, which avoids information skew when converting from the plain text to the embedded vector. For this purpose, a text-embedding process was performed. By deploying phrase embedding, we can extract a numerical representation of a phrase that can encapsulate its semantic content. Sentence-embedding methods are usually based on pretrained deep neural language models, such as GPT [40], BERT [41], and SBERT [42].

**Figure 2:**
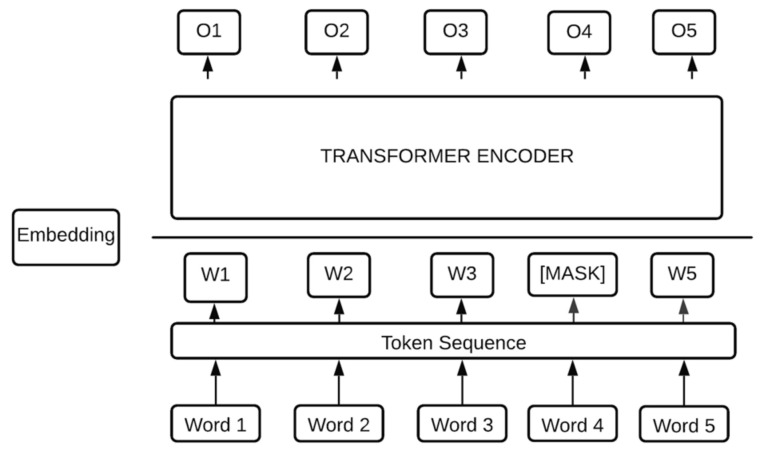
BERT model [41]. The raw texts are fed into the model to predict the binary label. It takes the CLS token as input, and then passes it to the transformer block, which results in embedded vectors.

BERT [41] is a transformer model for NLP that was developed by Google. Its model architecture is a multilayer bidirectional transformer encoder, designed to pretrain deep bidirectional representations from a plain text by jointly conditioning on a left–right context. It is trained on a large corpus of unlabeled text, which enables it to learn words and sentence-level information to perform efficient language masking. It is useful for tasks such as entity recognition, speech tagging, question-answering, word prediction, and text classification. Its model architecture is composed of multihead attention layers. There are variations in the model architecture; however, its basic modeling consists of 12 Transformer blocks, 768 hidden layers, and 12 self-attention heads. The total number of parameters for the pretrained model is 110 M. Figure 2 shows the BERT architecture.

**Figure 3:**
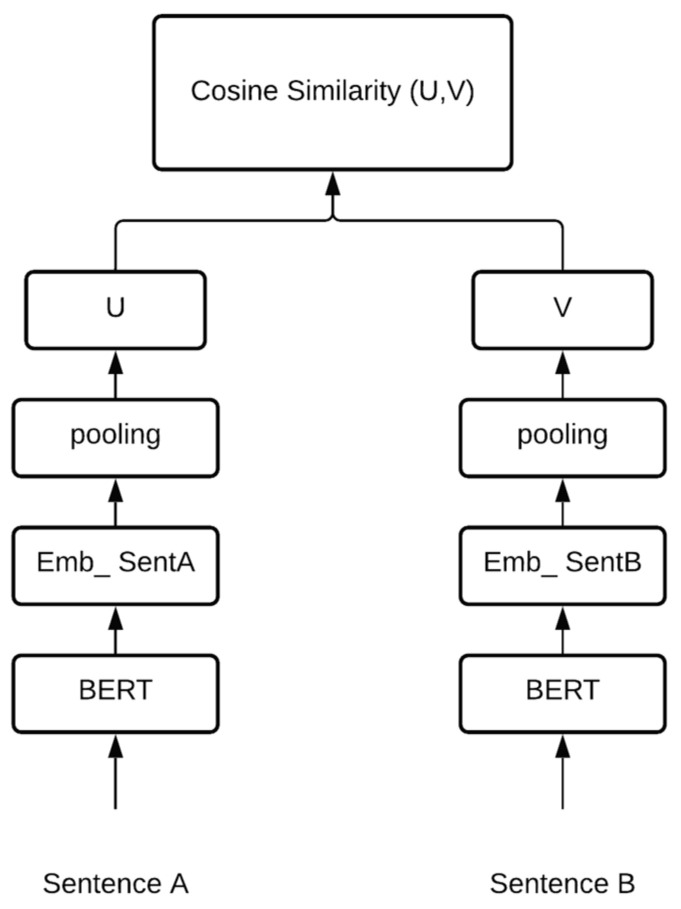
SBERT architecture [42]. Having Sentence A and Sentence B as input, the embeddings, u and v, are produced after BERT pooling. The similarity of these embeddings is computed using cosine similarity.

The BERT model is not an option for semantic similarity searches or clustering. This observation has inspired the realization of a new sentence-embedding model. SBERT [42] is a modified model based on BERT that uses network structures to derive semantically meaningful sentence embeddings performing cosine similarity and other tasks, such as semantic textual similarities, semantic searches, or paraphrase mining. It integrates the Siamese network with a pretrained BERT model. SBERT adds a pooling operation to the output of BERT. This pooling layer allows us to create a fixed-size representation for the variable-length input phrases. Figure 3 shows the SBERT architecture.

The “similarity” refers to the common characteristics between two instances, a magnitude that is calculated by the distance between them, and an appropriate threshold is established to decide whether they are similar or not [43]. This metric is favorable for data mining, information retrieval, and text-matching processes. In this case, it has been applied to find information that matches the relevant points of the description. This research uses cosine similarity to compare documents by extrapolating plain text to an n-dimensional vector space, calculating the cosine of the angle formed between two vectors. This is calculated with Equation (2), where *P* is the document weight, *n* represents the number of terms, *d* is the document, and *q* is the query [44]:

(2) $$\mathrm{SimCos}(d,q)\;=\;\frac{\Sigma \left(P_{\left(n,d\right)}\times P_{\left(n,q\right)}\right)}{\sqrt{\Sigma {\left(P_{\left(n,d\right)}\right)}^{2}\times \Sigma {\left(P_{\left(n,q\right)}\right)}^{2}{}^{}}}$$

It is worth mentioning that the semantic features were extracted on the basis of linguistic research conducted by experts in aphasia and geriatrics [11]. The keywords searched in the text are shown in Table 2, while the text’s central ideas are presented in Table 3.

The use of keywords within the text demonstrates that the central theme is being inferred in a general way. By finding these words in the descriptions, we assume that the task is being performed correctly, and this means that the patient is referring to the scene of the image, and is not digressing into another topic. As has been shown, AD patients present correlations between the changes in the information in the description of the image and the scores of the concepts [12]. For this reason, we calculated the number of keywords used in the description in order to assess if there is an inference to the image, and how frequently it is made.

Table 3 describes the key points of the image action sequence and divides it into headings with regard to the attention needed to emphasize it during the description. We take advantage of these points to make a specific evaluation of the given explanation. By adding the semantic load during text embedding, it is possible, with Equation (2), to evaluate the similarities between these main ideas and what is claimed in the text. This avoids a textual search of these ideas, allowing a wider spectrum in the comparison by quantifying the extent to which these subthemes are addressed. This not only facilitates the calculation of the similarities by fragmenting the search into short sentences, but it is also beneficial because it avoids biased information.

To have an overview of what is described, we have also performed a cosine similarity calculation to the ground-truth description. The magnitude of similarity achieved gives us an approximation of the performance of the task.

### 3.5. Importance of the Characteristics

In terms of the main purposes of this research, they are not only to extract linguistic features from neuropsychological assessments, but also to quantify the usefulness of these features in the task of dementia classification. For this purpose, we have applied the correlation method to evaluate the bidirectional association between the MMSE score and the class labels. We focused on using Spearman’s correlation method [45]. The correlation coefficient obtained was −0.789, with a significance of $2.26561\times 10^{-118}$, and a statistical power equal to 1. Note that the MMSE score and the denomination of dementia present a strong negative correlation, with high statistical power. This means that the higher the score achieved on the test, the more likely it is that a patient can be ruled out as someone diagnosed with cognitive impairment or dementia. In this sense, a feature highly related to MMSE has a higher probability of being a useful feature for the classification tasks of dementia.

The mutual information measures the relationship between two random variables, even if the relationship is not linear. It is based on the concept of the relevance probability of each attribute, which is measured employing a permutation test, allowing it to discard irrelevant variables, as well as to order by importance those which are relevant [46]. It is calculated by Equation (3) and is based on the Shannon entropy [47] of the pair, “(*X, Y*)”, where *p* is the joint probability distribution function of *X* and *Y*, and *f* and *g* are the marginal probability distribution functions of *X* and *Y*, respectively.

(3) $$I(X,Y)=\Sigma_{x\epsilon X}\Sigma_{y\epsilon Y}\;P(X,Y)\log\;\frac{P_{\left(X,Y\right)}}{f_{\left(X\right)}g_{\left(Y\right)}}$$

It is important to point out that the mutual information score measure is defined from (0–1) [48], where two cases are possible:

1. I(*X*,*Y*) = 0: the variables are independent, in other words, they do not share information;
2. I(*X*,*Y*) > 0: there is an association between X and Y, so they share information.

**Figure 4:**
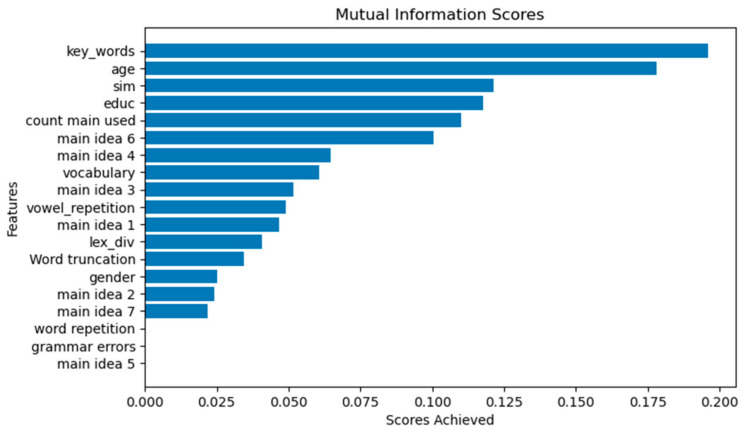
Histogram of the scores achieved by each of the characteristics concerning the MMSE score in the mutual information analysis.

The scores achieved in the mutual information method show that the most related characteristic is the one called “keywords”, which are extracted from the semantic characteristics (Figure 4). The data with the greatest relevance for the naming of dementia are “age” and “education”, as well as some of the semantic characteristics. This reaffirms the idea of applying this type of feature to the classification model.

### 3.6. Automatic Classifiers

In terms of machine learning, a classification is a discriminative approach that predicts the categorical outcomes from a set of input variables. Given samples from a specific class, the goal is to predict the probability of an element drawn from the same class [48].

In this work, we have applied K-nearest Neighbors, random forest, support vector machine, and an artificial neural networks as classification methods. The choice of the methods used is based on their results in the state-of-art methods for similar tasks.

#### 3.6.1. K-Nearest Neighbors

**Figure 5:**
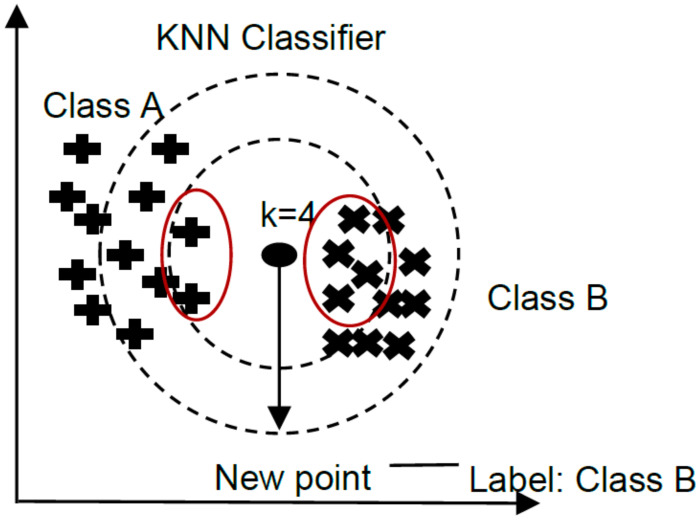
KNN classification method.

K-nearest neighbors (KNN) bases its logic on calculating the distances between points, since any data can be projected as a point in an n-dimensional space, where n is the number of input attributes, Figure 5. The algorithm distinguishes between the most similar observations, considering the points closest to each other as the same class [49]. The rationale is based on the assumption that features belonging to the same label tend to overlap. To perform the prediction of unlabeled data, a number, k, of neighbors is defined. The unlabeled data is classified by the number of k neighbors, defined as the nearest in terms of the Euclidean distance.

#### 3.6.2. Random Forest

**Figure 6:**
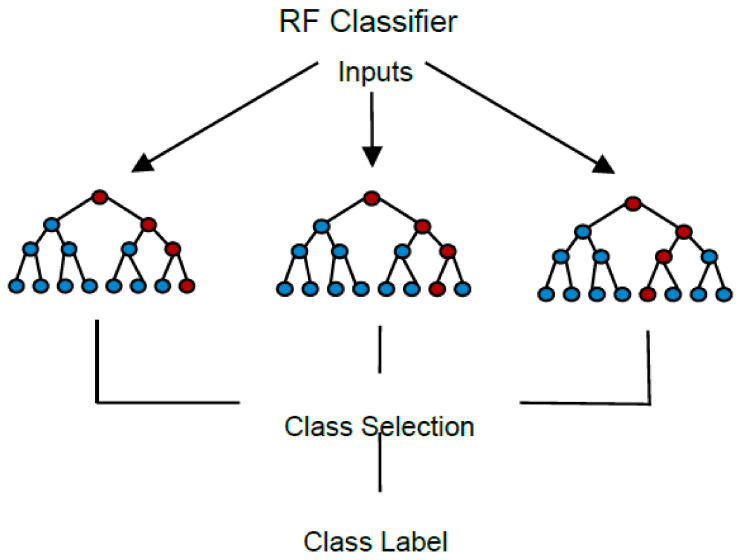
RF classification method.

Random forest (RF) is an ensemble methodology that is formed by a collection of decision trees, Figure 6. To be built, it needs to specify the number of base trees and tree-specific parameters, such as the leaf size, the depth, and the splitting criteria [50]. Each decision tree in the ensemble acts as a base classifier to determine the class label of a new entry, and the algorithm takes the mean value of the outputs of the decision trees to define a final label.

#### 3.6.3. Support Vector Machine

**Figure 7:**
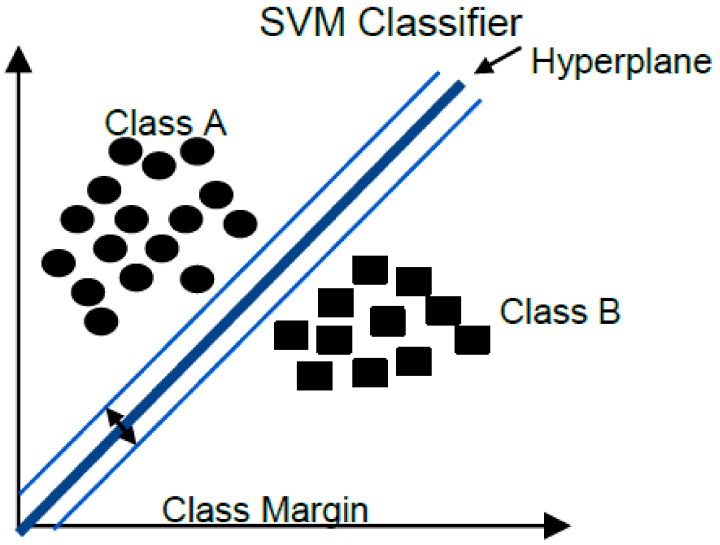
SVM classification method.

Support vector machine (SVM) seeks to delimit semispaces for data separation from a basic set of points that can help to identify and establish a boundary; these points are called “support vectors” [51]. The model is built by defining the boundary of each class, placing a hyperplane, and setting a class margin. Once a boundary is defined, we proceed to check if the data is within the boundary. The class label is determined by the position of the observation with respect to the hyperplane, Figure 7.

#### 3.6.4. Artificial Neural Network

**Figure 8:**
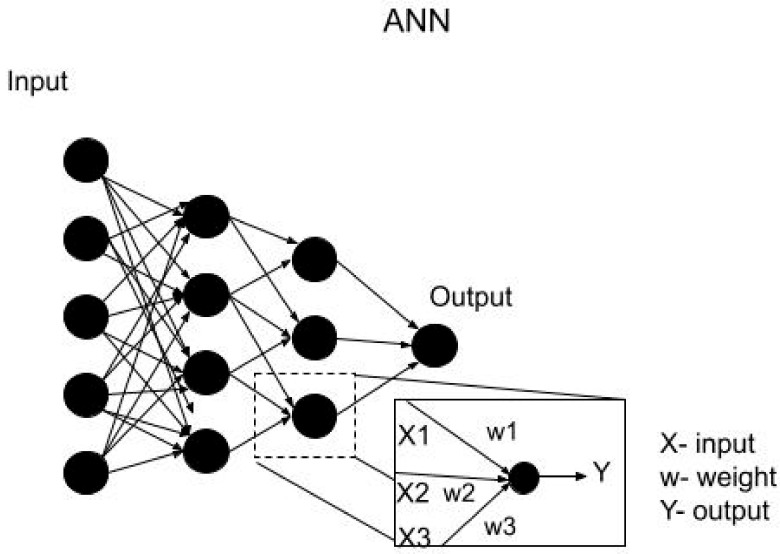
ANN classification method.

This approach tries to emulate the biological process of a neuron. Its basic architecture can be described as a graph, whose nodes are connected to each other, forming a network where the relationships between the input and class labels are defined to generate a prediction [52], Figure 8. In the core, a neural unit takes a weighted sum of its inputs, with an additional term called “bias”. Posteriorly, a nonlinear activation function is applied to each unit, which is, in fact, the final output. The model learning is carried out by adjusting the weights of each of the neural units using the backpropagation algorithm.

#### 3.6.5. Model Setups 

A binary classification was made, which aimed to discriminate people with or without dementia. As is seen in Table 4, we used six models from the SKLearn python package [53]. In addition, we performed a neural network classification from the TensorFlow library [54].

**Table 4:** Models and their hyperparameters.

Model	Hyperparameters
KNN	K = 30
Random Forest	Trees = 100, max depth = 6
SVM Linear	kernel = ‘linear’
SVM Polynomial	kernel = ‘poly’, degree = 2
SVM Precomputed	kernel = ‘precomputed’
SVM RBF	kernel = ‘rbf’
Artificial Neural Network	Please see Figure 9

To define the classifier’s hyperparameters, different aspects were taken into account. In the case of K-nearest neighbor (KNN), the elbow method [55] was implemented to define the number of K neighbors, while, for the random forest, a cross-validation was performed. On the other hand, for the support vector machine (SVM), standard kernels were used to map the observations. To find the best configuration for the artificial neural network (ANN), we used the Autokeras library [37].

### 3.7. Proposed Methodology

**Figure 10:**
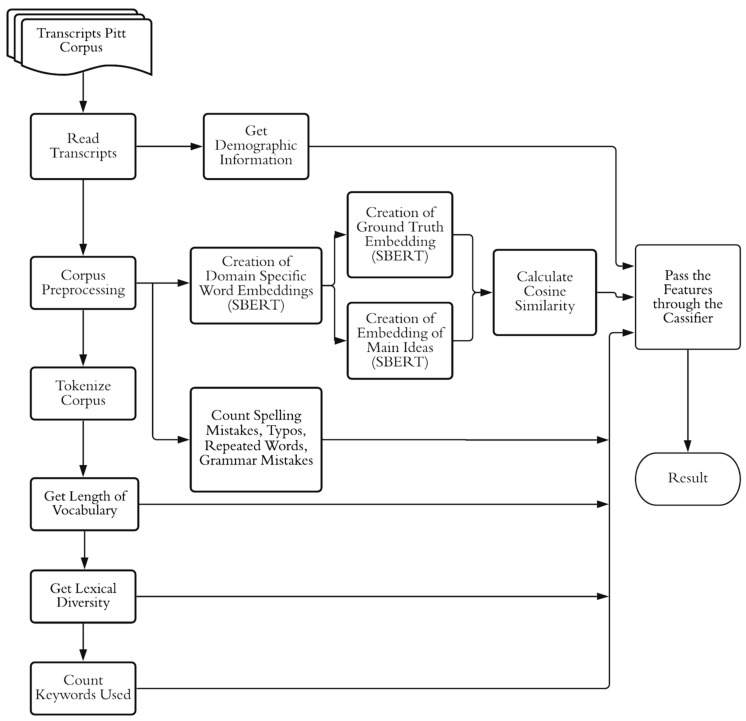
Flowchart of proposed methodology.

We propose the methodological process shown in Figure 10. As mentioned in Section 3.1, the database provides an accumulation of the speech samples classified in HC and AD subjects. These transcripts briefly provide information about the test subject, the researcher’s cues and interventions, as well as the description provided during the test.

The plain text extracted from the description is generated by preprocessing, which consists of extracting only the participant’s response, removing the CHAT symbology from this text ascribed by the researchers and the punctuation marks, and converting the text to lowercase.

Once the plain text is ready to be processed, the linguistic features described above are extracted.

At the end of the feature extraction, we generated a data frame where all the linguistic properties obtained in the text are stored. To evaluate the performance of the proposed classification methods, cross-validation was performed for each method to obtain the summary statistics. The extruded features have also been varied in order to weight and record the observations.

## 4. Results

In order to get an accurate overview of the performance of our proposed methodology, two strategies were followed: the first employed the metrics commonly used in similar studies: the accuracy, precision, recall, and *F1-score* [56] metrics. The metrics mentioned above are defined in terms of the correctly estimated values, namely, the true positives (*TP*) and true negatives (*TN*), as well as the incorrect predictions, namely, the false positives (*FP*) and false negatives (*FN*) [52].

- *Accuracy*: Represents the percentage of correct predictions compared to the total. In our context, the accuracy refers to the ratio between the correct predictions of dementia and all the analyzed cases:
  (4) $$Accuracy=\frac{TP\;+\;TN}{TP\;+\;FP\;+\;FN\;+\;TN}\;\times \;100\;\;$$
- *Precision*: This is also called the “true-positive rate”. In our case, the precision gives us the ratio between patients classified as suffering from dementia, according to our method, and patients who actually have dementia:
  (5) $$Precision=\frac{TP}{TP+FP}\;\times \;100\;$$
- *Recall*: The percentage of positive dementia cases that were correctly identified:
  (6) $$Recall=\frac{TP}{TP+\;FN}\;\times \;100\;\;\;\;\;$$
- *F1-score*: The harmonic mean between the *Recall* and the *Precision*. This metric eliminates the bias caused by having unbalanced data, such as in our dataset (Table 1):
  (7) $$F1-score=\frac{2}{\frac{1}{Precision}\;\times \;\frac{1}{Recall}}\;\times 100\;\;\;$$

The second strategy uses k-fold cross validation to decrease the possible bias in the performance measurements that are due to the small number of samples in our dataset (550). For each classification model, the data was partitioned into 10 folds, splitting the data into training and testing. The 10-fold cross method was iterated 10 times.

Table 5 and Table 6 show the statistics of each metric per classification algorithm. Table 5 is the result of the classification conducted exclusively with lexical and syntactic characteristics, where the amount of vocabulary used, the lexical diversity, the spelling errors, the typos, and the grammatical errors are obtained. These characteristics are used in [20,21,22,23,24,25,26,27,28,29]. In this experimentation, an average accuracy of 71% was achieved using a KNN, being the lowest average classification value, and the highest accuracy of 75% was achieved when using the polynomial and precomputed SVM. The average precision values achieved were 68−80%, with the KNN having the highest percentage.

We aim to add semantic indicators to strengthen the model. Table 6 shows the performances of the classification algorithms when adding the extracted semantic features to the model, which, in this case, are the keyword search in the text, the cosine similarity calculation with the main ideas proposed in Table 3, and the similarity calculation concerning the description considered to be our ground truth. The percentages achieved in the evaluation metrics increased, reaching an average accuracy of 78% when using an ANN. Nevertheless, the polynomial SVM reached higher percentages in the remaining metrics.

**Figure 11:**
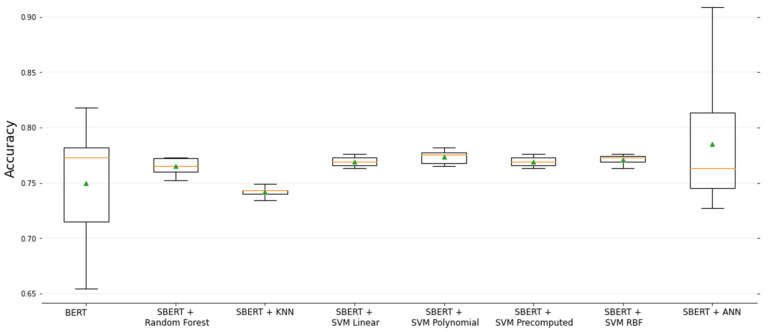
10-fold cross-validation comparison in terms of accuracy.

In order to compare the performance of our proposed methodology with state-of-the-art methods [35], in terms of the accuracy rate, we describe, through the 10-fold iterations, the classification performances in Figure 11. It is important to mention that the BERT test was performed under the same data conditions, except for the use of manual transcriptions, and only having the corpus embedding available.

The ANN shows the best performance in many folds. However, the variability of its classification rate is a warning about its reliability. The SVM classifiers maintain a normal classification rate, with an average classification rate of 77%, indicating that, in general terms, it is the most viable classifier for this task.

## 5. Discussion

The approach used in this research work was inspired by the linguistic investigations carried out by specialists in this clinical area [11,12]. Throughout the realization of this work, we encountered challenges in obtaining the proposed features that are due to the prevalence of sparse and noisy data. The preprocessing techniques reduced the noise; however, a combination of techniques to further remove it is required. Implementing NLP techniques in the database increases the search spectrum of the patterns and features. However, there is still an error factor that is difficult to determine.

On the other hand, the use of pretrained models minimizes the computational load needed to perform the text-embedding process, which provides a semantic interpretation of the text because of the architecture implemented in these models and the richness of the data with which it is pretrained.

Concerning the extraction of the semantic features, which is our object of interest, the degree of the usefulness of the features was calculated. The application of the mutual information method reveals a degree of dependence between the proposed features and the MMSE score, with the features corresponding to the use of keywords, age, similarity of the description with respect to the ground truth, and academic degree being the ones with higher scores in the evaluation. With this, we can infer that the semantic and demographic information have higher weights in the classification model. At this point, it is important to note that, by experimenting with a different selection of features, the lexical and syntactic linguistic features have lower performances than those performed by adding semantic features. On the basis of the experiments, the classification methods with the best scores in the metrics were the second-order polynomial SVM model and the ANN model, reaching average accuracies of 77% and 78%, respectively, at their maximum values. Misdiagnosis translates directly into time lost to pause the progression of this disease. Thus, it is important to maintain a low number of false positives over false negatives for validation. Therefore, we used the result obtained in the F1 score, and we obtained an average of 80% for this metric.

## 6. Conclusions

Linguistic features, as postulated in the research mentioned here, are elements that reflect a cognitive deficit, and, specifically, aphasia. It is clear that it is not possible to use this aspect as a determinant in the clinical classification of dementia; however, it can be used as a supportive test to discard cases of probable dementia, especially in its early stages. We argue that a basic model for the denomination of dementia could be deprecated on the MMSE score if the lexical, syntactic, semantic, and demographic features are used. The results obtained from our experimental test show that the SBERT approach through cosine similarity is adequate for generating semantic features. Moreover, we have shown that the configuration by using SBERT + SVM performed better in each metric than the rest of the models, except for the configuration, SBERT + ANN, which had better accuracy rates in some of the folds. Through the comparison between the lexical-syntactic model and the semantic-syntactic model, we have proven that the implementation of semantic features to the model increases the metrics rates by approximately 3%.

While the proposed methodology performed acceptably well for dementia discrimination, continuing the search for more relevant linguistic or clinical features could be reflected in improvements in the performance tasks. It has been shown that the semantic features improve the performances of classification models because of the approach with which the diagnostic evaluation is developed. Looking for other syntactic and semantic features that will provide us with information about the semantic memories of test subjects, as well as the implementation of an ensemble model, will be discussed in future work.

## Figures and Tables

**Figure 9:**
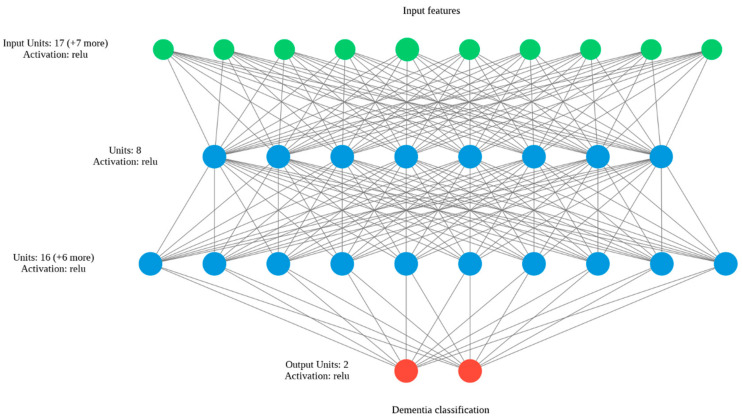
Artificial neural network architecture.

**Table 1:** AD and HC group comparisons of demographics and MMSE mean scores.

	Subjects	Gender	Age	MMSE	Education Years
HC	243	90M/153F	46–81	29	8–21
AD	307	118M/189F	49–90	20	6–20

**Table 2:** Keywords used to capture possible semantic information [11].

	Keywords	
boy	jar	overflowing
girl	plate	spilling
woman	sink	asking
kitchen	stool	unconcerned
window	water	indifferent
cabinet	taking	stealing
cookies	cookie	wobbling
counter	falling	handing
curtain	drying	mother
dishes	washing	sister
faucet	doing	brother
floor		

**Table 3:** Main ideas extracted on the basis of the thematic coherence classification system in [12].

Topic Concept	Analysis	Main Idea
Cookie thief scene	A clause or noun phrase that identifies a global description of the content of a sequence of utterances.	Children stealing cookies
Character activity	Those subtopic sequences encompassing the activities carried out by each of the characters.	Woman doing dishes Girl reaching for a cookie Woman not noticing Boy on stool
Additional information	An additional layer in the hierarchy of the description.	Sink overflowing Stool falling

**Table 5:** Average results obtained in the experiment using only lexical-syntactic features.

Model	*Accuracy*	*Precision*	*Recall*	*F1 Score*	*Time (Seg.)*
Random Forest	0.74	0.78	0.74	0.76	**0.01**
KNN	0.71	**0.80**	0.63	0.71	0.04
SVM Linear	0.74	0.78	**0.76**	**0.77**	**0.01**
SVM Polynomial	**0.75**	0.79	**0.76**	**0.77**	**0.01**
SVM Precomputed	0.74	0.78	**0.76**	**0.77**	**0.01**
SVM RBF	0.75	0.79	0.75	0.76	**0.01**
Artificial Neural Network	0.72	0.68	0.72	0.70	5.35

**Table 6:** Average results were obtained in the experiment using lexical, syntactic, and semantic characteristics.

Model	*Accuracy*	*Precision*	*Recall*	*F1 Score*	*Time (Seg.)*
Random Forest	0.76	0.79	0.78	0.78	**0.01**
KNN	0.74	**0.81**	0.70	0.75	0.04
SVM Linear	0.77	0.80	0.79	0.79	**0.01**
SVM Polynomial	0.77	0.80	**0.80**	**0.80**	**0.01**
SVM Precomputed	0.77	0.80	0.79	0.79	**0.01**
SVM RBF	0.77	**0.81**	0.78	0.79	**0.01**
Neural Network	**0.78**	0.73	0.79	0.76	8.43
